# Factors influencing healthcare providers’ behaviours in deprescribing: a cross-sectional study

**DOI:** 10.1080/20523211.2024.2399727

**Published:** 2024-09-16

**Authors:** Chee Tao Chang, Huan-Keat Chan, Ewilly Jie Ying Liew, Muhammad Radzi Abu Hassan, Jason Choong Yin Lee, Wee Kooi Cheah, Xin Jie Lim, Philip Rajan, Siew Li Teoh, Shaun Wen Huey Lee

**Affiliations:** a School of Pharmacy, Monash University Malaysia, Bandar Sunway, Malaysia; b Clinical Research Centre Hospital Raja Permaisuri Bainun, Institute for Clinical Research, National Institute of Health, Ministry of Health Malaysia, Ipoh, Malaysia; c Clinical Research Centre Hospital Sultanah Bahiyah, Institute for Clinical Research, National Institute of Health, Ministry of Health Malaysia, Alor Setar, Malaysia; d School of Business, Monash University Malaysia, Bandar Sunway, Malaysia; e Perak Pharmaceutical Services Division, Ministry of Health Malaysia, Tanjung Rambutan, Malaysia; f Clinical Research Centre Hospital Taiping, Institute for Clinical Research, National Institute of Health, Ministry of Health Malaysia, Taiping, Malaysia; g Department of Medicine, Hospital Taiping, Ministry of Health Malaysia, Taiping, Malaysia

**Keywords:** Medication discontinuation, polypharmacy, geriatric conditions, patient safety, healthcare policy

## Abstract

**Introduction:**

Deprescribing serves as a pivotal measure to mitigate the drug-related problem due to polypharmacy. This study aimed to map the factors influencing healthcare providers’ deprescribing decision using the Behaviour Change Wheel framework and develop an innovative conceptual model to support deprescribing practice.

**Methods:**

A cross-sectional online survey targeting doctors and pharmacists was conducted to assess the influence of various factors on healthcare providers’ comfort in recommending deprescribing. The conceptual model was formulated, based on the existing deprescribing framework and the Behaviour Change Wheel. The model’s robustness was scrutinised through Partial Least Squares Structural Equation Modeling (PLS-SEM), and model-fitting indices were employed to obtain the best-fit model.

**Results:**

A total of 736 responses were analysed with the final best-fit model consisting of 24 items in 5 constructs (*R*^2^: 0.163; SRMR: 0.064; rho_c: 0.750–0.862; AVE: 0.509–0.627) and three independent factors. Based on the results, we proposed that deprescribing could be promoted through strategies aimed at enhancing healthcare providers internal capabilities such as knowledge levels, when patients’ condition deteriorated and previous experiences with adverse events of drugs. Organisational support in providing such educational opportunities is important, with the empowerment of patient and healthcare providers through policy enhancements, guideline development, and effective communication.

**Conclusion:**

The deprescribing behaviours of healthcare professionals are influenced by an intricate interplay of patient, prescriber, and system factors. Enhancing deprescribing practices necessitates a comprehensive strategy that encompasses providers and patients’ education, the development of structured deprescribing guidelines, the implementation of deprescribing support tools, and the enhancement of communication between healthcare providers.

## Introduction

The practice of polypharmacy, defined as the concurrent use of five or more medications, is increasingly prevalent among older adults, raising critical concerns about the potential for inappropriate medication practices that could compromise both patient safety and the effectiveness of treatments (Masnoon et al., 2017). This concern is magnified by the demographic shift towards an aging population, highlighting the urgent need for refined strategies to optimise medication use among seniors to enhance their health and well-being (Rochon et al., 2021).

Polypharmacy consistently leads to an increased risk of potentially inappropriate medications (Abdulah et al., 2018; Chang et al., 2023b). According to a systematic review, the prevalence of polypharmacy and potentially inappropriate medications in the Malaysian older population was 49.5% and 28.9%, respectively (Chang et al., 2021). Polypharmacy was significantly associated with adverse outcomes, including increased readmissions and emergency room revisits after discharge (Liang et al., 2022). Furthermore, polypharmacy has been linked to a 1.5–2 times higher likelihood of recurrent falls in older adults (Ming & Zecevic, 2018). Ahmed et al. reported that polypharmacy was 2.3 times more likely to be associated with adverse drug reactions (Ahmed et al., 2014).

These underscore the critical need for effective management strategies to address polypharmacy and its associated risks in the older population. By employing strategies aimed at streamlining and optimizating medication use, healthcare professionals can address the adverse consequences associated with polypharmacy (Rochon et al., 2021). An important aspect of addressing polypharmacy is deprescribing, a process that involves the careful reduction or cessation of medications, with the aim of enhancing patient outcomes (Thompson & Farrell, 2013).

According to a systematic review, deprescribing interventions could effectively reduce the number of drugs or doses taken by older patients (Omuya et al., 2023). A Canadian study reported that a deprescribing intervention consisting of a pharmacist-led medication review significantly reduced the number of medications taken by nursing home residents (Balsom et al., 2020). Similarly, a study conducted among older adults from the end of hospitalisation through post-acute care demonstrated that a deprescribing intervention reduced the number of medications by approximately 15% and decreased patient exposure to potentially inappropriate medications (Vasilevskis et al., 2023). Additionally, a randomised controlled trial among older individuals approaching the end of life found that the number of medications and monthly medication costs were significantly reduced following STOPPFrail-guided deprescribing interventions (Curtin et al., 2019).

Despite being recognised as beneficial, deprescribing presents significant challenges for healthcare professionals (Kua et al., 2019a; Nadarajan et al., 2017) due to time constraints, the pressure to achieve therapeutic targets, patient resistance, safety concerns, the absence of evidence-based guidelines and specialised training in deprescribing (Djatche et al., 2018; Linsky et al., 2019; Tangiisuran et al., 2021).

Understanding the barrier and challenges experienced by healthcare providers and patients in their deprescribing journey can help policy makers in creating targeted intervention. For instance, healthcare professionals’ reluctance to adopt less aggressive goals can be achieved through customising performance indicators and treatment objectives to suit the needs of individual patients, rather than adhering rigidly to conventional threshold values (Linsky & Zimmerman, 2018).

The Behaviour Change Wheel (BCW) offers a comprehensive approach to understanding and influencing behaviour changes necessary for effective deprescribing (Michie et al., 2011). It has been instrumental in identifying motivating factors for deprescribing among healthcare providers and adapting deprescribing practices to local contexts (Bai et al., 2022). By aligning the motivating factors with specific deprescribing strategies, the BCW has facilitated the customisation of deprescribing initiatives to meet the unique needs to fit local contexts, proving invaluable in engaging stakeholders and actions in supporting deprescribing initiatives in academic settings (Farrell et al., 2023). This study aims to refine the existing conceptual model of deprescribing by integrating insights from prior models with findings from this research, adding depth and context to the understanding of these elements and their impact on deprescribing practices.

## Methods

### Study design, setting, and participants

This was a cross-sectional study, conducted from November 2022 to March 2023 across 13 states and 3 federal territories in Malaysia. To be eligible for the study, participants had to be a registered doctor or pharmacist, actively practicing in a healthcare facility in Malaysia. The study encompassed a diverse range of medical specialties, including but not limited to internal medicine, cardiology, endocrinology, gastroenterology, infectious disease, palliative care, neurology, and physiatry.

Data collection was conducted through an online survey distributed by email through the Ministry of Health and relevant professional societies, personal contacts, and snowball sampling.

### Sample size

Sample size was estimated using the rule-of-thumb calculation for SEM, recommending 10 cases per parameter, resulting in a minimum sample of 580 to accommodate the 58 parameters in the model (Bowen & Guo, 2011). In this study, we targeted a minimum of 600 samples.

### Study instrument

The survey utilised a validated questionnaire from a previous study (Linsky et al., 2016), with permission granted for its use and modifications. Face validation was conducted by a panel consisting of one geriatrician, one internal medicine physician, and two academicians. Their feedback was incorporated during a meeting with all study investigators, leading to necessary modifications to ensure the questionnaire’s relevance and feasibility for our target population.

The questionnaire, administered in English, contains 23 questions, assessing how deprescribing factors influenced their inclination to recommend discontinuation of a medication in a regular month using a five-point Likert scale (‘much less likely’ [1] to ‘much more likely' [5]). Additionally, the questionnaire assessed the overall comfort level of deprescribing was measured using a 10-point sliding scale, where 0 signifies ‘Not at all comfortable' and 10 indicates ‘completely comfortable’. The questionnaire was pilot tested with a group of 10 doctors and pharmacists for readability, feasibility, and ease of completion. This process led to the removal of four items related to respondents’ perception towards healthcare providers, effectively focusing the questionnaire more directly on deprescribing. The refined items were then analysed using principal component analysis, which was used to inform the development of the conceptual model.

### Statistical methods

Confirmatory factor analysis was used to assess the validity of the latent variables on the present study sample using a cut off loadings greater than 0.40 (Broen et al., 2015; Stevens, 2001) and cross-loadings less than 0.40. Subsequently, we employed the Partial Least Squares Structural Equation Modeling (PLS-SEM) analysis to assess the associations between latent variables (constructs) and participants’ comfort with deprescribing.

To evaluate model validity, we examined convergent validity through the average variance extracted (AVE) threshold of >0.5 and discriminant validity was assessed using the heterotrait-monotrait (HTMT) ratio of correlations of <0.90 (Hair et al., 2019). To validate the predictive relevance of the structural model, the Stone-Geisser’s *Q*² criteria was used. Model comparison was conducted using various fitting indices to select the most suitable model. Statistical analysis was carried out in SPSS v28.0 (IBM SPSS) and the SEM modelling was implemented using SmartPLS 4 (Oststeinbek, Germany), using a significance threshold of *p* < .05 for all two-sided statistical tests.

### Conceptual model development

**Figure 1.**
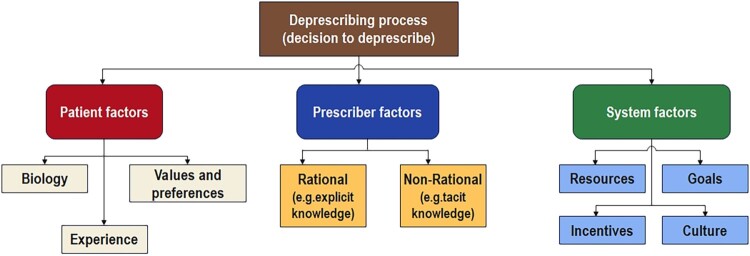
Deprescribing conceptual model by Linsky et al. (2019). Note: Authorised reproduction of this Figure has been granted.

The development of the conceptual model for this study was informed by the foundational model proposed by Linsky et al. (2019) (Figure 1). In the original model by Linsky and colleagues, they identified three pivotal factors in deprescribing: patient factors (such as biology, experiences, values, and preferences), prescriber factors (including knowledge, both rational and tacit, as well as values and preferences), and systemic factors (resources, incentives, goals, and culture) (Linsky et al., 2019).

**Figure 2.**
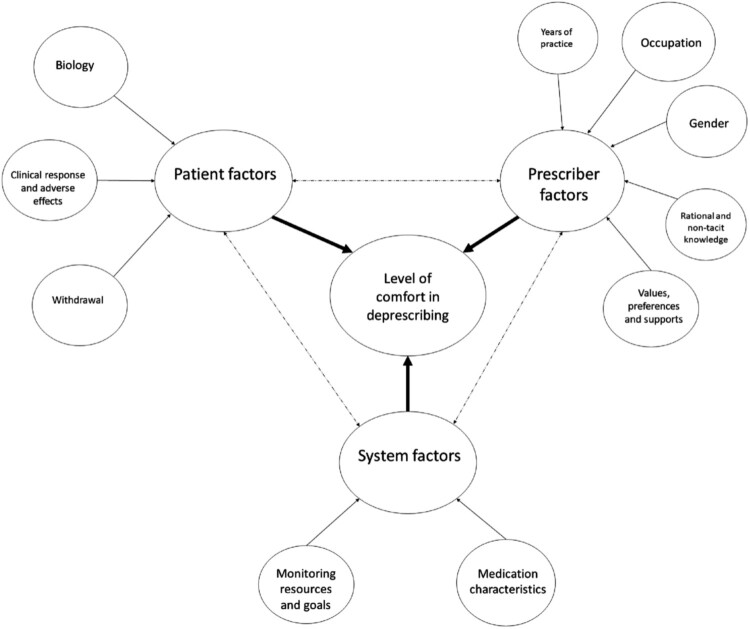
Hypothesised conceptual model.

In our study, we aim to advance the hypothesis that addressing deprescribing effectively requires a multifaceted strategy that not only incorporates but also expands upon the three core domains initially identified by Linsky et al. (2019). In our model, we account for a broader range of factors. These enhancements include an in-depth examination of prescriber factors, which assess the healthcare provider’s experience, professional role, gender, and cognitive insights, as well as their personal values, preferences, and the support mechanisms at their disposal. On the patient side, our model further explores biological considerations, clinical reactions, potential adverse effects, and the complexities surrounding the cessation or reduction of medications. System factors in our expanded model scrutinise the inherent properties of medications, the availability and efficacy of monitoring resources, and the strategic objectives underpinning medication management practices (Figure 2).

### Ethical considerations

Prior to participation, all participants provided written informed consent. The study received approved from the Medical Research and Ethics Committee (KKM/NIHSEC/P21-1469(4)) and adhered to the relevant guidelines and regulation with the Declaration of Helsinki.

## Results

### Sample characteristics

A total of 764 responses were recorded. Twenty-eight responses were excluded as: 11 were non-medical personnel, 10 incomplete response and 7 which did not consent. The final sample included 736 responses, with a median age of 35 years old (IQR: 8 years); 488 (66.3%) were female, and 450 (61.6%) worked in public hospitals. Demographic data are provided in Table 1.

**Table 1.** Demographic characteristics of respondents (*n* = 736).

Characteristics	Frequency	Percentage
Age (median, IQR)	35.0	8
**Gender**		
Male	248	33.7
Female	488	66.3
**Type of setting**		
Public hospitals	450	61.1
Public health clinics	143	19.4
Public administrative	51	6.9
Private clinic	22	3.0
Academic	20	2.7
Retail pharmacy	15	2.0
Private hospital	12	1.6
Others	23	3.1
**Occupation**		
Doctor	316	42.9
Pharmacists	420	57.1
**Years of experience in current setting (median, IQR)**	7.0	9.0
**Years of experience overall (median, IQR)**	10.0	8.0

### Factors affecting deprescribing

The factor analysis incorporated 26 items designed to evaluate factors influencing deprescribing practices. These items were categorised into seven distinct constructs, each representing a crucial aspect of the deprescribing process (Table 2). The constructs include: medication characteristics; biology; clinical response and adverse effects of current medications; withdrawal events after deprescribing; values, preferences, and supports; rational explicit and non-rational tacit knowledge; monitoring resources and goals. These constructs demonstrated robust reliability, with a Cronbach’s alpha coefficient of 0.85, indicating a high degree of internal consistency among the items within each construct.

**Table 2.** Factors which influence doctors and pharmacists’ decision in recommending medication discontinuation (*n* = 736).

No.	Factors	Mean (SD)	Factor loading
	Medication characteristics		
F1	Medication can treat more than one condition	3.10 (1.19)	0.622
F2	Medication used for long time	3.00 (1.09)	0.756
F11	Medication is expensive	3.24 (1.05)	0.515
	**Biology**		
F3	Significant physical health conditions	3.97 (0.87)	0.727
F4	Significant mental health conditions	3.91 (0.91)	0.864
F5	Presence of cognitive impairment	3.99 (0.89)	0.841
F6	Limited life expectancy (<6 months)	3.73 (1.06)	0.513
	**Clinical response and adverse effects of current medications**		
F7	Objective clinical response of the medications	2.32 (1.02)	0.637
F8	Subjective clinical response of the medications	2.27 (0.91)	0.719
F12	Objective findings of non-life threatening adverse effects	3.76 (0.88)	0.713
F13	Subjective experience of adverse effects	3.82 (0.85)	0.657
	**Withdrawal events after deprescribing**		
F9	Physiologic adverse drug withdrawal event	3.53 (1.00)	0.672
F10	Doctors’ concern symptoms return if medication stopped	3.46 (1.07)	0.794
F14	Patients’ concern symptoms return if medication stopped	3.40 (1.04)	0.756
	**Values, preferences, and supports**		
F15	Patients’ preference to continue the medication	3.29 (1.05)	0.685
F16	Patient’s good understanding of health condition	3.75 (0.97)	0.765
F17	Good adherence to the regimen	3.38 (1.07)	0.769
F22	Social support available for patients	3.59 (1.08)	0.783
F23	Strong relationship with patients	3.31 (0.99)	0.539
	**Explicit and tacit knowledge**		
F18	Formal education	3.62 (1.15)	0.668
F19	Continuing education	4.22 (0.81)	0.843
F20	On-the-job experience	4.36 (0.72)	0.819
F21	Prior successful experience with medication discontinuation	4.04 (0.90)	0.705
	**Monitoring resources and goals**		
F24	Monitoring support when medications discontinued	3.69 (0.93)	0.581
F25	Clinical guidelines with relaxed treatment targets	3.89 (0.78)	0.489
F26	Pressure to meet performance measures	3.02 (0.91)	0.633

Note: Question: During a typical month, how did the following influence on your likelihood to recommend discontinuation of a medication? Measured using 5-point Likert ‘much less likely' to ‘much more likely'

## Initial structural model

The confirmative factor analysis showed good structural validity for the proposed latent constructs, except for medication characteristics and withdrawal events which had suboptimal rho_C values (rho_C = 0.569 and rho_C = 0.003 respectively). As the model fit was poor and reliability was low (*R*^2^: 0.155; SRMR: 0.110; rho_c: 0.003–0.862; AVE: 0.169–0.611), two items (F10 and F11) were excluded from the initial model.

Two constructs, i.e. values, preferences, supports [F15, F16, F17, F22, F23 (*β* = 0.043, *p* = .269)] and withdrawal effects after deprescribing [F9, F14 (*β* = −.048, *p* = .292)] were not significantly associated with deprescribing comfort and was dropped in our final model.

## Final structural model

**Figure 3.**
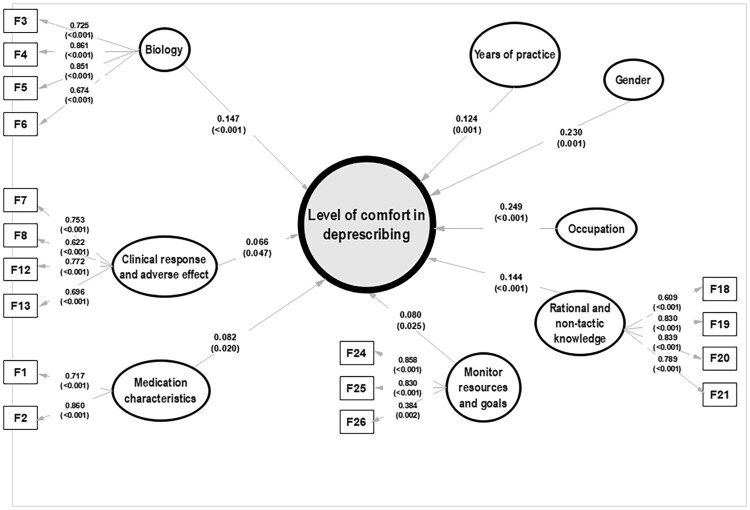
Final structural model.

The final model achieved a better fit, comprising 24 items in 5 constructs (*R*^2^: 0.163; SRMR: 0.064; rho_c: 0.750–0.862; AVE: 0.509–0.627) along with three independent predictors (Figure 3). Overall, healthcare professionals’ comfort to recommend deprescribing was affected by (i) patient biological condition, (ii) their own knowledge level, (iii) the clinical response and adverse effects, (iv) medication characteristics, and (v) monitoring resources and goals. This is in addition to the three independent factor, namely: gender (*β* = 0.230, *p* = .001), occupation (*β *= 0.272, *p* < .001), and years of practice (*β* = 0.124, *p* = .001) of the practitioners.

The final model’s predictive performance, indicated by a *Q*² value of 0.130, showed a predictive advantage over the baseline linear model. Additionally, the best-fit model exhibits lower Root Mean Square Error (RMSE) and Mean Absolute Error (MAE) values (1.798 and 1.384, respectively) in comparison to the naive linear model (with RMSE and MAE values of 1.799 and 1.395).

**Figure 4.**
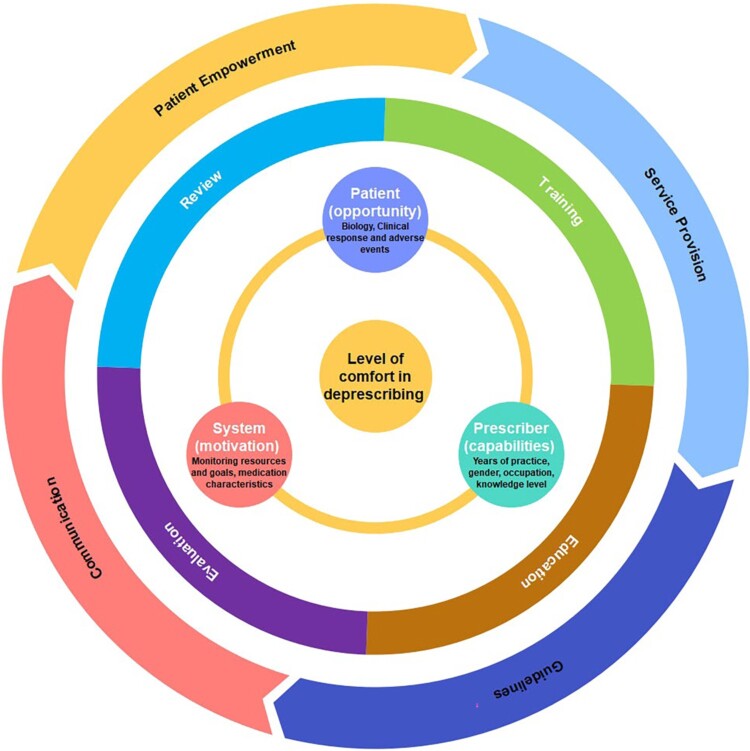
Innovative deprescribing wheel.

Drawing from our findings, we developed the Innovative Deprescribing Wheel (IDW), rooted in the principles of the BCW, which is designed to foster deprescribing behaviours (Figure 4). The IDF is structured into three concentric layers, each targeting key factors in the deprescribing process.

At its core, the IDW focusses on the inherent capabilities of healthcare professionals, such as their experience, expertise, gender, and professional role, which can impact their approach to deprescribing. Additionally, patient-related factors could present opportunities for deprescribing, by emphasising the importance of aligning patients’ values and priorities, and closely monitoring their clinical responses and potential adverse events from medication adjustments. Moreover, system-level factors play an important role in influencing healthcare professionals’ deprescribing decisions, shaped by the availability of resources in their setting and the medication characteristics being utilised.

The middle layer addresses the organisational context, specifically the healthcare setting such as clinics or hospitals where healthcare professionals work. This layer highlights the importance of having a supportive environment, ensuring that healthcare professionals have sufficient training, alongside structured processes which are essential to support deprescribing processes.

The outermost layer expands to encompass the broader healthcare system, focuses on initiatives to support deprescribing efforts. These initiatives include improving communication between all stakeholders, developing clear deprescribing guidelines, enhancing mechanisms for service provision, and fostering strategies that empower patients in their healthcare journey. Together, these layer presents a holistic framework designed towards encouraging and facilitating deprescribing practices, emphasising the interconnected roles of individual capabilities, organisational support, and systemic initiatives.

## Discussion

The present study describes a refined deprescribing conceptual framework (IDW), that merges insights from our findings with the Behavior Change Wheel (BCW). The augmented model (IDW) not only builds upon a previously established model by Linsky et al. (2019) but enriches it by emphasising the crucial role of medication characteristics and situational factors that influence healthcare professionals’ decision-making processes and spotlights key motivators for deprescribing.

Our analyses revealed that prescriber attributes, particularly their knowledge, experience, and gender emerged as paramount in influencing deprescribing. These findings resonate with the core of the IDW, where the prescriber’s expertise will determine their willingness to deprescribe. As reported previously, healthcare providers have a higher propensity to deprescribe when they are confident of their knowledge and experience, underscoring the necessity for ongoing continuous education and training in deprescribing (Farrell et al., 2023; Heinrich et al., 2023; Silva Almodóvar et al., 2024).

In addition, patient-related factors, particularly their health status significantly affect deprescribing willingness. This aligns with previous research (Drenth-van Maanen et al., 2020; Goyal et al., 2020), suggesting a targeted approach towards patients with poorer health status and those at higher risk of adverse drug reactions. This tailored strategy underscores the importance of a patient-centered approach in optimising medication regimen. While system-related factors appeared less influential, they remain integral to a holistic deprescribing strategy. In this study, we noted the role of medication characteristics and available resources in supporting deprescribing decisions (Fialová et al., 2018).

Therefore, the IDW advocates for enhanced healthcare professional education and training to strengthen their deprescribing capabilities. Educational programmes can be developed to facilitate learning activities aimed at updating and expanding healthcare professionals’ knowledge and skills in the context of medication discontinuation (Cervero & Gaines, 2015). This should also involve practical, skill-based training that fosters interprofessional collaboration, encouraging a culture of conducive to safe and effective deprescribing (Anderson et al., 2014; Heinrich et al., 2023; Linsky & Zimmerman, 2018). Thus, it’s essential to establish a formal, structured system for transferring information among patients, caregivers, and healthcare providers. Such a mechanism ensures that critical medication information is consistently shared across different care settings – from hospitalisation through to discharge and into primary care follow-up. This comprehensive exchange facilitates a cohesive approach to medication management, ensuring that decisions regarding deprescribing are informed, timely, and reflect the continuum of care for each patient (Fried et al., 2017).

In terms of medication management, our findings point towards the necessity of a systematic medication review, prioritising the reassessment of medications for patients with multiple comorbidities or those at an increased risk of medication-related issues. This approach is critical for aligning treatment with individual patient needs and minimising potential risks associated with polypharmacy. Some medications that could be targeted for deprescribing include those that are prescribed to address multiple health conditions simultaneously, such as alpha blockers, antidepressants, and antipsychotics (Chang et al., 2023a). These multi-purpose medications can be complex to manage and may pose potential risks when used for extended periods.

While contemporary clinical practice guidelines extensively cover medication prescribing, there exists a noticeable gap in providing comprehensive guidance for deprescribing. This absence of structured deprescribing guidelines emerges as a significant barrier within the deprescribing process, as highlighted by our research and corroborated by other studies. Recognising this gap, the Innovative Deprescribing Wheel (IDW) framework stresses the critical need for the development of explicit deprescribing guidelines. Such guidelines aim to furnish healthcare professionals with a systematic and standardised approach, offering clear instructions on evaluating the appropriateness of deprescribing specific medications for particular patient groups, thereby facilitating safer and more effective medication management practices (Heinrich et al., 2022; Kua et al., 2019b).

Equally critical to the deprescribing process is the active involvement of patients. Engaging patients in discussions about their medication management allows for the leveraging of their unique perspectives and experiences, which can be instrumental in identifying medication-related issues, including the use of potentially inappropriate medications. Therefore, empowering patients is of paramount importance in the deprescribing process. Enhancing patient empowerment can be achieved by boosting their self-efficacy, for instance, through the provision of decision aids. Such tools have been shown to significantly increase patients’ willingness to participate in decisions about discontinuing medications, thereby playing a vital role in promoting a more patient-centered approach to medication management (Martin & Tannenbaum, 2017).

Within healthcare institutions, the adoption of computerised alert systems into electronic medical records is advocated as a significant strategy to support the deprescribing process. These digital alert systems are engineered to proactively notify healthcare providers of potential opportunities for medication discontinuation, informed by the patient’s clinical outcomes and adverse reactions to medications (Coe et al., 2021). Serving as crucial decision-support mechanisms, these alerts are designed to highlight critical moments when deprescribing may be beneficial, ensuring healthcare professionals receive timely and relevant information. Beyond merely flagging potential deprescribing cases, these systems play a pivotal role in enhancing patient safety by reducing the likelihood of adverse drug events. They achieve this by enabling healthcare providers to promptly re-evaluate and adjust medication plans in alignment with evolving patient health statuses, thus ensuring more responsive and patient-centered care (Page et al., 2017).

At the policy level, the IDW calls for the development of clear deprescribing guidelines and the integration of patient engagement strategies into the deprescribing process. Enhancing patient self-efficacy and ensuring robust communication and information exchange among healthcare teams are pivotal for the successful implementation of deprescribing interventions (Drenth-van Maanen et al., 2020). Similarly, patients with limited life expectancies present an opportunity for deprescribing (Anderson et al., 2014; Goyal et al., 2020). The IDW framework underscored the significance of evaluating a patient’s overall health condition as a patient-centered approach to tailor a treatment plan to the individual’s specific needs.

## Strengths and limitation

This study offers several strengths. Firstly, it adopts a rigorous approach to explore doctors’ and pharmacists’ perceptions regarding medication discontinuation among older adults. This approach was validated through an iterative research process and confirmed using PLS-SEM. The introduction of the IDW model is also a significant advancement, providing deeper insights into the mechanisms of deprescribing. The IDW serves as a valuable conceptual model for the development of future deprescribing interventions and its alignment with the mission of the international working group of the United States Deprescribing Research Network (Silva Almodóvar et al., 2024) underscores the relevance and potential impact on the global health initiative towards improving medication safety especially in older population.

Nevertheless, there are some limitations to this study. Our survey respondents included 80.5% who worked in public hospitals and clinics, which collectively provide 70% of the healthcare needs in the country. These respondents included physicians and pharmacists working across different fields of practice including geriatrics, internal medicine among others distributed across various states within Malaysia. Nevertheless, as our study used snowball sampling, this may have introduced some sample bias, which can make it difficult to estimate the sampling error. As such, we urge caution in the interpretation of these findings, as our results may have underrepresented the responses from the private settings. To build on this research, future studies should consider stratified sampling and include the qualitative perspectives of healthcare professionals to attain a more comprehensive understanding of deprescribing practices.

## Conclusion

The study intricately mapped the perspectives of doctors and pharmacists on medication discontinuation for older patients within the Behaviour Change Wheel (BCW) framework, revealing that healthcare professionals’ comfort with deprescribing is shaped by a complex mix of factors. The Integrated Deprescribing Wheel (IDW) model that emerged from this analysis is a comprehensive strategy that emphasises the importance of education of healthcare providers, patients, and caregivers. This strategy should be further supported by the development of thorough deprescribing guidelines and the promotion of effective communication channels. Together, these elements aim to foster a well-rounded approach to deprescribing, ensuring that it is conducted safely, effectively, and in a manner that is sensitive to the needs and circumstances of older patients.

## Supplementary Material

STROBE_checklist_filled.doc

## Data Availability

All data to reproduce the tables and figures in the manuscript and Supplemental Materal can be obtained with reasonable request from the corresponding author.
